# *In Vitro* Activity of KBP-7072 against 536 Acinetobacter baumannii Complex Isolates Collected in China

**DOI:** 10.1128/spectrum.01471-21

**Published:** 2022-02-09

**Authors:** Renru Han, Li Ding, Yang Yang, Yan Guo, Dandan Yin, Shi Wu, Peiyuan Zhi, Demei Zhu, Qingmei Liu, Xiaojuan Tan, Yuanju Zhu, Jay Zhang, Li Li, Fupin Hu

**Affiliations:** a Institute of Antibiotics, Huashan Hospital, Fudan University, Shanghai, China; b Key Laboratory of Clinical Pharmacology of Antibiotics, Ministry of Health, Shanghai, China; c KBP Biosciences Co. Ltd., Jinan, Shandong, China; d KBP Biosciences USA Inc., Princeton, New Jersey, USA; College of New Jersey

**Keywords:** carbapenem-resistant A. baumannii, colistin, KBP-7072, omadacycline, tigecycline

## Abstract

Acinetobacter baumannii has emerged globally as a difficult-to-treat nosocomial pathogen and become resistant to carbapenems, resulting in limited treatment options. KBP-7072 is a novel semisynthetic aminomethylcycline, expanded spectrum tetracycline antibacterial agent with completed phase 1 clinical development studies. This study aimed to evaluate the *in vitro* activity of KBP-7072 and several comparators against clinical A. baumannii isolates collected from China. A collection of 536 A. baumannii clinical isolates were isolated from 20 hospitals across 13 provinces and cities in China between 2018 and 2019. Antimicrobial susceptibility testing of 12 antimicrobial agents was performed utilizing the broth microdilution method recommended by CLSI. KBP-7072 has shown active antibacterial activity against 536 A. baumannii isolates. It inhibited the growth of all isolates at 4 mg/liter, including 372 carbapenem-resistant isolates, 37 tigecycline MIC ≥ 4 mg/liter isolates, and 138 omadacycline MIC ≥ 4 mg/liter isolates. Compared with other expanded spectrum tetracyclines, KBP-7072 (MIC_90_, 1 mg/liter) outperformed 2-fold and 4-fold more active against 536 A. baumannii isolates than tigecycline (MIC_90_, 2 mg/liter) and omadacycline (MIC_90_, 4 mg/liter). KBP-7072 was as equally active as colistin (MIC_90_, 1 mg/liter, 99.4% susceptible). Doxycycline (33.4% susceptible), gentamicin (31.3% susceptible), meropenem (30.6%, susceptible), imipenem (30.2% susceptible), ceftazidime (27.8% susceptible), piperacillin-tazobactam (27.2% susceptible), and levofloxacin (27.2% susceptible) showed marginally poor antibacterial activity against tested isolates according to CLSI breakpoints, except for minocycline (73.7% susceptible). KBP-7072 is a potential alternative agent for the treatment of infection caused by A. baumannii, including carbapenem-resistant species.

**IMPORTANCE** It is reported that A. baumannii has emerged as an intractable nosocomial pathogen in hospitals especially when it develops resistance to carbapenems and other antibiotics, which limits treatment options and leads to high mortality. In February 2017, the WHO published a list of ESKAPE pathogens designated “priority status” for which new antibiotics are urgently needed. Therefore, the epidemiological surveillance and new therapeutic development of A. baumannii must be strengthened to confront an emerging global epidemic. KBP-7072 is a novel, expanded spectrum tetracycline antibacterial and has demonstrated good *in vitro* activity against recent geographically diverse A. baumannii isolates collected from North America, Europe, Latin America, and Asia-Pacific. This study has shown excellent *in vitro* activity of KBP-7072 against clinical A. baumannii isolates collected from different regions of China, regarded as supplementary to KBP-7072 pharmacodynamics data, which is of great significance, as it is promising an alternative treatment in CRAB isolates infections in China.

## INTRODUCTION

Infections caused by Acinetobacter baumannii, including pneumonia, bloodstream infections, urinary tract infections, skin and skin soft tissue infections, burn and surgical wound infections, endocarditis, meningitis, and osteomyelitis, commonly occur in hospitalized patients who have undergone medical treatments involving indwelling hardware, such as mechanical ventilators, intravascular catheters, urinary catheters, and drainage tubes (1–5). It is reported that A. baumannii has emerged as an intractable nosocomial pathogen in hospitals, especially when it develops resistance to carbapenems and other antibiotics, which limits treatment options and leads to high mortality (1, 6–9). In February 2017, the WHO published a list of pathogens for which new antibiotics are urgently needed. Within this broad list, ESKAPE (Enterococcus faecium, Staphylococcus aureus, Klebsiella pneumoniae, A. baumannii, Pseudomonas aeruginosa, and Enterobacter species) pathogens were designated “priority status” (5). The epidemiological surveillance and new therapeutic development of A. baumannii must be strengthened to confront an emerging global epidemic.

**FIG 1:**
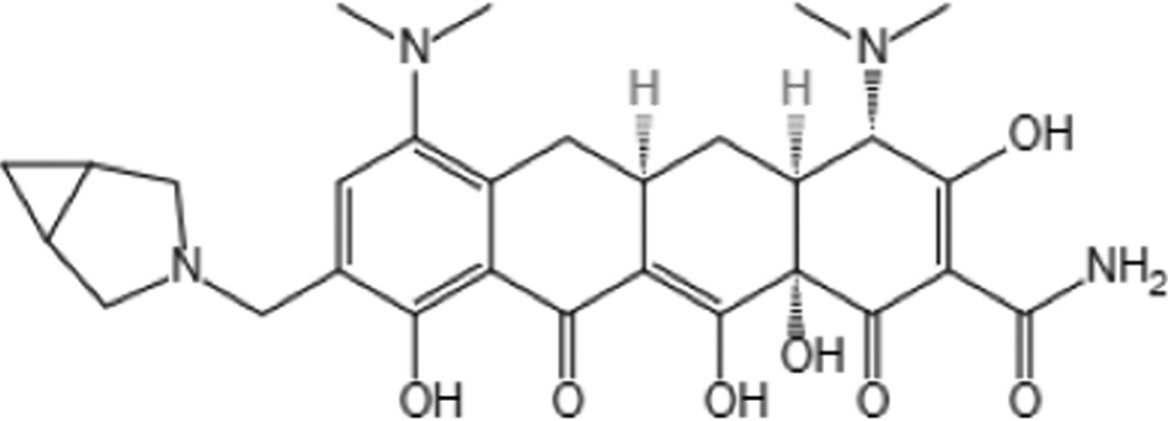
KBP-7072 compound structure.

KBP-7072 (Fig. 1) is a novel, broad-spectrum, semisynthetic aminomethylcycline, expanded spectrum tetracycline antibacterial in clinical development for acute bacterial skin and skin structure infections (ABSSSI), community-acquired bacterial pneumonia (CAP), and complicated intraabdominal infections (cIAI) (10). It inhibits the normal function of the bacterial ribosome and has demonstrated good *in vitro* activity against recent geographically diverse, molecularly characterized, and drug-resistant A. baumannii isolates, which can overcome many common tetracycline resistance mechanisms (10).

KBP-7072 has been developed for oral and intravenous formulations and completed phase 1 clinical development studies for safety, tolerability, pharmacokinetics (ClinicalTrials.gov identifier NCT02454361), and multiple ascending doses in healthy subjects (ClinicalTrials.gov identifier NCT02654626) in December 2015 (10). The pharmacokinetics/pharmacodynamics (PK/PD) index area under the concentration-time curve (AUC)/MIC correlated well with efficacy (11). The PK results in animal models are consistent with single and multiple ascending dose studies in healthy volunteers and confirm the suitability of KBP-7072 for once-daily oral and intravenous administration in clinical studies (12). In this study, we evaluated the *in vitro* activity of KBP-7072 and comparators utilizing broth microdilution against 536 A. baumannii clinical isolates isolated from 20 hospitals across 13 provinces and cities in China between 2018 and 2019.

## RESULTS

### *In vitro* activity of KBP-7072 and comparators against 536 A. baumannii isolates.

**FIG 2:**
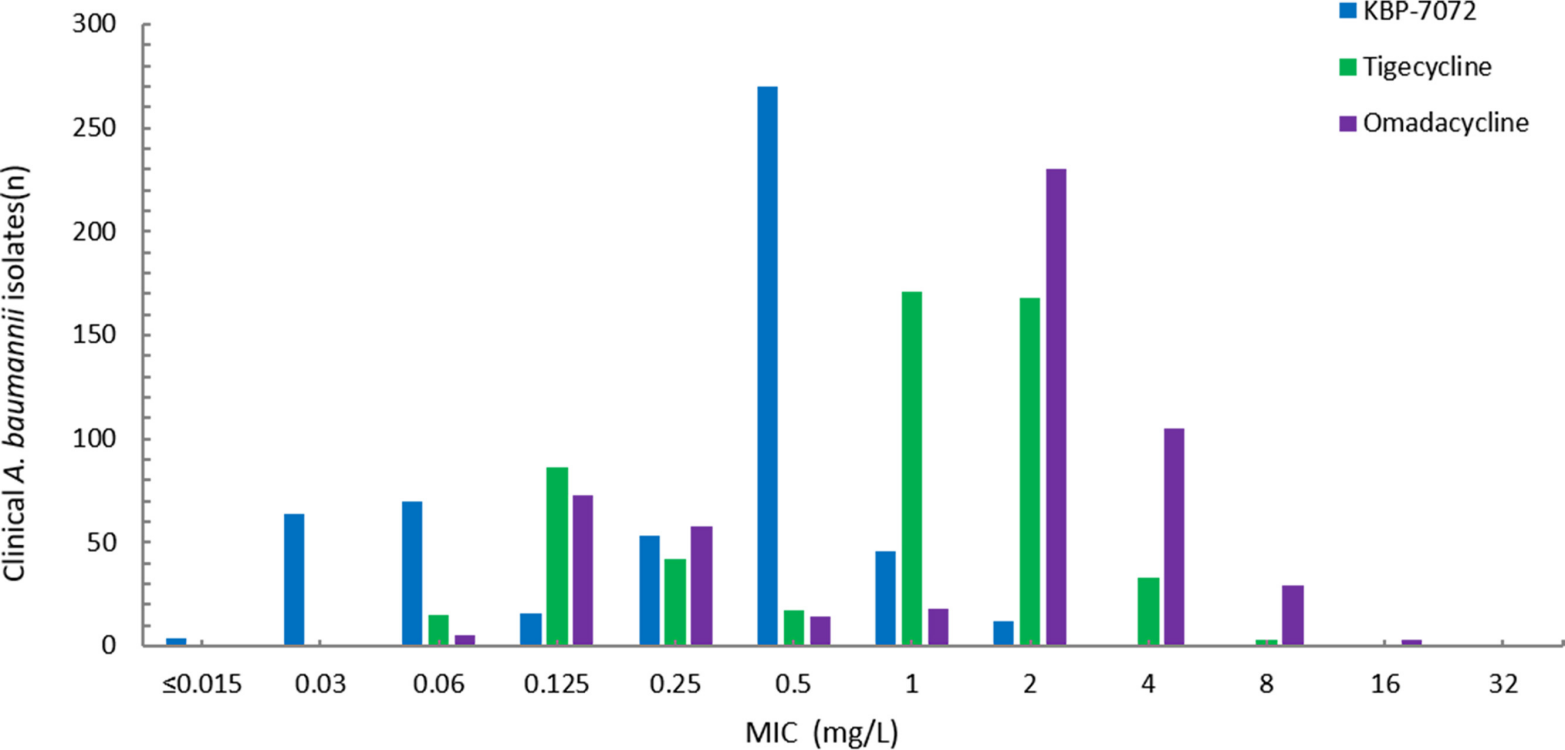
MIC distribution of KBP-7072, tigecycline, and omadacycline for 536 A. baumannii isolates.

KBP-7072 has shown active antibacterial activity against 536 A. baumannii isolates with MIC_50_ and MIC_90_ of 0.5 mg/liter and 1 mg/liter, respectively, and 4 mg/liter of KBP-7072 can inhibit the growth of all tested isolates, including carbapenem-resistant isolates (Table 1 and Fig. 2). Compared with other expanded spectrum tetracyclines, the MIC_90_ of KBP-7072 (MIC_90_, 1 mg/liter) was 2-fold and 4-fold lower than that for tigecycline (MIC_90_, 2 mg/liter) and omadacycline (MIC_90_, 4 mg/liter). Moreover, tigecycline and omadacycline need to reach 16 mg/liter and 32 mg/liter *in vitro*, respectively, which can inhibit the growth of all tested isolates. Colistin has also shown excellent antibacterial activity against A. baumannii isolates *in vitro* with MIC_50_ at 0.5 mg/liter and MIC_90_ at 1 mg/liter, consistent with KBP-7072. Doxycycline (33.4% susceptible), gentamicin (31.3% susceptible), meropenem (30.6%, susceptible), imipenem (30.2% susceptible), ceftazidime (27.8% susceptible), piperacillin-tazobactam (27.2% susceptible), and levofloxacin (27.2% susceptible) showed marginally poor antibacterial activity against tested isolates according to CLSI breakpoints. Overall, the other antimicrobial agents showed slightly *in vitro* activity against tested isolates, except for tigecycline, omadacycline, minocycline (73.7% susceptible), and colistin (99.4% susceptible).

**TABLE 1:** *In vitro* activities of KBP-7072 and comparators against 536 A. baumannii isolates

Antimicrobial agents	MIC (mg/liter)				R%	S%
	MIC ranges	MIC_50_	MIC_90_	Mode		
KBP-7072	≤0.015–4	0.5	1	0.5		
Omadacycline	0.06–32	2	4	2		
Tigecycline	0.06–16	1	2	1		
Doxycycline	≤0.06–>128	32	64	32	66	33.4
Minocycline	≤0.06–64	4	8	4	8	73.7
Gentamicin	0.125–>128	>128	>128	>128	68.1	31.3
Ceftazidime	0.5–>128	128	>128	>128	72	27.8
Imipenem	≤0.06–>128	64	>128	64	69.2	30.2
Meropenem	≤0.06–>128	32	128	64	69	30.6
Piperacillin-tazobactam	≤0.06–>128	>128	>128	>128	72	27.2
Levofloxacin	≤0.06–128	8	32	8	62.5	27.2
Colistin	0.125–8	0.5	1	0.5	0.6	99.4

### *In vitro* activity of KBP-7072 and comparators against 372 CRAB isolates.

**FIG 3:**
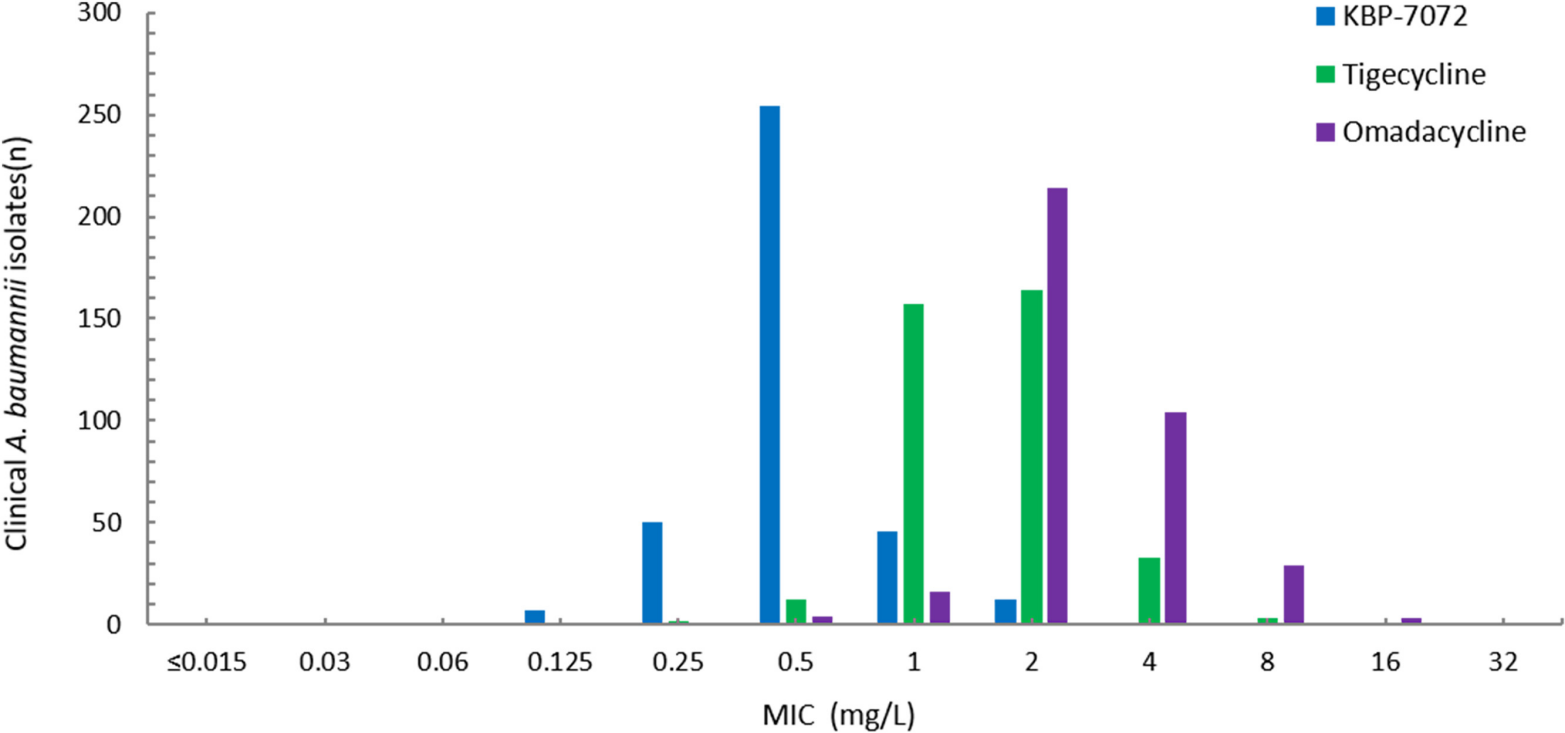
MIC distribution of KBP-7072, tigecycline, and omadacycline for 372 carbapenem-resistant A. baumannii isolates.

**FIG 4:**
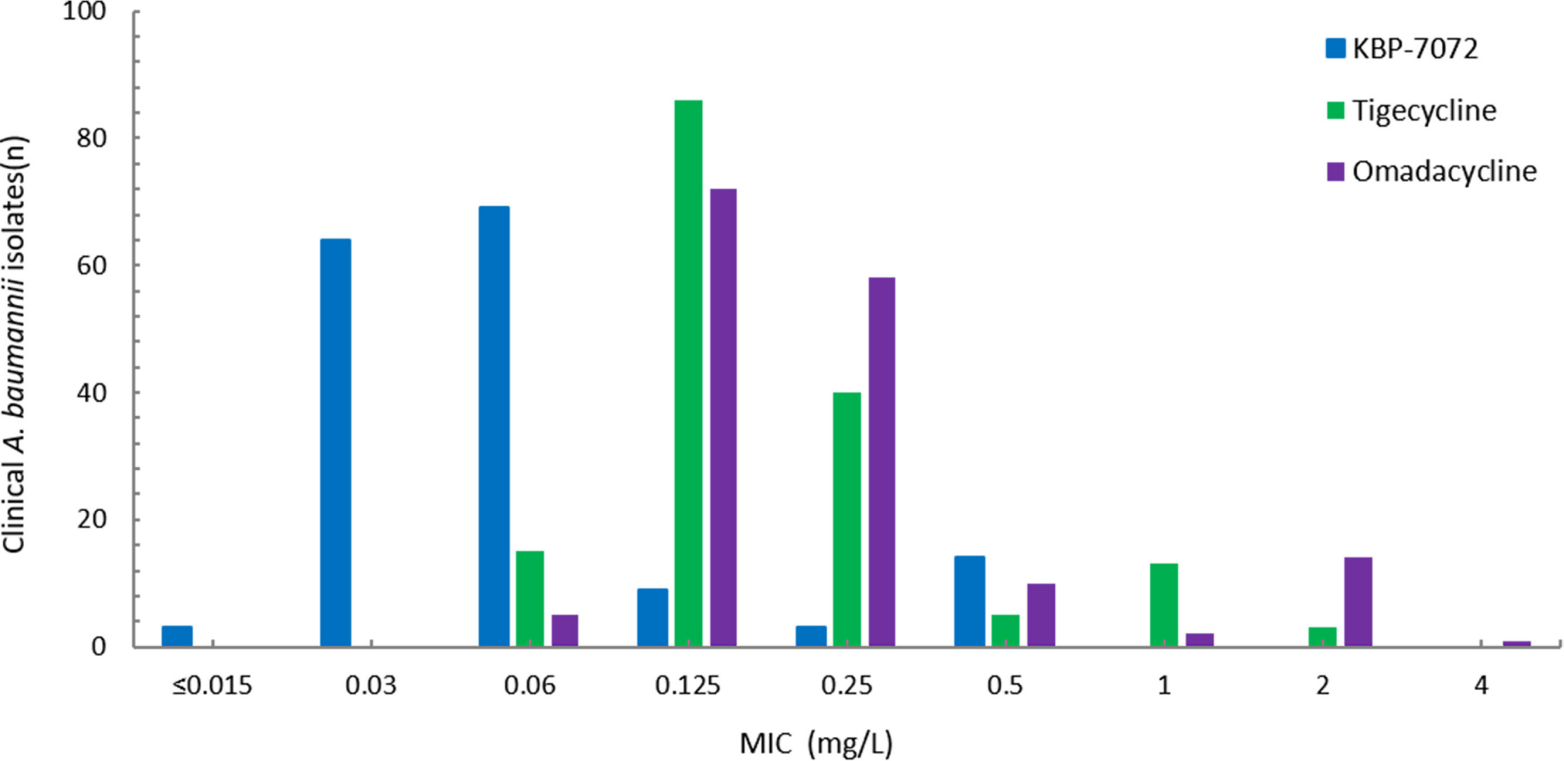
MIC distribution of KBP-7072, tigecycline, and omadacycline for 162 carbapenem-susceptible A. baumannii isolates.

In this study, 372 of tested isolates (69.4%) were carbapenem-resistant *A. baumannii* (CRAB), defined as, resistant to at least one of carbapenem antibiotics (imipenem or meropenem), and 164 (30.6%) were susceptible or intermediate to imipenem and meropenem (Fig. 3 and 4). The MIC_50_ and MIC_90_ of KBP-7072 against CRAB isolates were 0.5 mg/liter and 1 mg/liter, respectively. In comparison with tigecycline (MIC_90_, 2 mg/liter) and omadacycline (MIC_90_, 4 mg/liter), KBP-7072 demonstrated more significant antibacterial activity against CRAB isolates. Similarly, colistin (100% susceptible) has also shown excellent antibacterial activity with MIC_90_ at 0.5 mg/liter (Table 2). Other comparator agents, like doxycycline (6.7% susceptible), gentamicin (7.3% susceptible), ceftazidime (0.8% susceptible), piperacillin-tazobactam (0.3% susceptible), and levofloxacin (1.1% susceptible) were inactive against CRAB isolates with less than 8% susceptible, while minocycline showed some antibacterial activity with 65.1% susceptible. Notably, CRAB isolates usually exhibit multidrug-resistant characteristics. Carbapenem-susceptible or intermediate *A. baumannii* isolates were susceptible to most of tested antimicrobial agents (over 85% susceptibility). The MIC90 of KBP-7072, omadacycline, and tigecycline were 0.25, 1, and 0.5 mg/liter, respectively (Table 3).

**TABLE 2:** *In vitro* activities of KBP-7072 and comparators against 372 CRAB isolates

Antimicrobial agents	MIC (mg/liter)				R%	S%
	MIC ranges	MIC_50_	MIC_90_	Mode		
KBP-7072	≤0.015–4	0.5	1	0.5		
Omadacycline	0.125–32	2	4	2		
Tigecycline	0.25–16	2	2	2		
Doxycycline	≤0.06–>128	32	64	32	92.7	6.7
Minocycline	≤0.06–64	4	16	4	11.3	65.1
Gentamicin	0.5–>128	>128	>128	>128	92.5	7.3
Ceftazidime	2–>128	128	>128	>128	99.2	0.8
Imipenem	4–>128	128	>128	64	99.7	0
Meropenem	1–>128	64	128	64	99.5	0.3
Piperacillin-tazobactam	≤0.06–>128	>128	>128	>128	99.5	0.3
Levofloxacin	≤0.06–128	8	32	8	84.9	1.1
Colistin	0.125–2	0.5	0.5	0.5	0	100

**TABLE 3:** *In vitro* activities of KBP-7072 and comparators against 162 carbapenem-susceptible A. baumannii isolates

Antimicrobial agents	MIC (mg/liter)				R%	S%
	MIC ranges	MIC_50_	MIC_90_	Mode		
KBP-7072	≤0.015–0.5	0.06	0.25	0.06		
Omadacycline	0.06–4	0.25	1	0.125		
Tigecycline	0.06–2	0.125	0.5	0.125		
Doxycycline	≤0.06–64	0.125	2	≤0.06	4.3	95.1
Minocycline	≤0.06–16	≤0.06	1	≤0.06	0.6	94.4
Gentamicin	0.125–>128	0.5	>128	0.5	11.7	87
Ceftazidime	0.5–>128	4	8	4	9.3	90.1
Imipenem	≤0.06–2	0.25	2	0.25	0	100
Meropenem	≤0.06–2	0.25	1	0.25	0	100
Piperacillin-tazobactam	≤0.06–>128	1	32	≤0.06	8.6	89.5
Levofloxacin	≤0.06–64	0.125	8	≤0.06	10.5	87.7
Colistin	0.25–8	0.5	1	0.25	1.9	98.1

### *In vitro* activity of KBP-7072 and comparators against 37 tigecycline or 138 omadacycline MIC ≥ 4 mg/liter A. baumannii isolates.

KBP-7072 (MIC_90_, 2 mg/liter) and colistin (MIC_90_, 2 mg/liter) had a more active antibacterial activity against 37 A. baumannii isolates with tigecycline MIC ≥ 4mg/liter (MIC_90_, 8 mg/liter). Omadacycline has shown antibacterial activity against these 37 tested isolates with MIC_90_ of 8 mg/liter. There were three tested isolates with tigecycline MIC at 8 mg/liter (KBP-7072 at 2, 2, and 2 mg/liter, respectively; omadacycline at 8, 16, and 16 mg/liter, respectively) and one isolate with tigecycline MIC at 16 mg/liter (KBP-7072 at 4 mg/liter; omadacycline at 32 mg/liter). In addition, these tested isolates were all resistant to imipenem, meropenem, ceftazidime, piperacillin-tazobactam, and 97.3% resistant to doxycycline and levofloxacin, 94.6% to gentamicin, and 35.1% to minocycline (Table 4).

**TABLE 4:** *In vitro* activities of KBP-7072 and comparators against 37 tigecycline MIC ≥ 4 mg/liter A. baumannii isolates

Antimicrobial agents	MIC (mg/liter)				R%	S%
	MIC ranges	MIC_50_	MIC_90_	Mode		
KBP-7072	0.5–4	1	2	1		
Omadacycline	2–32	8	8	8		
Tigecycline	4–16	4	8	4		
Doxycycline	2–>128	64	128	64	97.3	2.7
Minocycline	1–32	4	16	4	35.1	54.1
Gentamicin	1–>128	>128	>128	>128	94.6	5.4
Ceftazidime	64–>128	>128	>128	>128	100	0
Imipenem	32–>128	128	>128	128	100	0
Meropenem	16–128	64	128	64	100	0
Piperacillin-tazobactam	128–>128	>128	>128	>128	100	0
Levofloxacin	4–128	16	64	16	97.3	0
Colistin	0.125–2	0.5	2	0.5	0	100

KBP-7072 (MIC_90_, 1 mg/liter) and colistin (MIC_90_, 1 mg/liter) had a more active antibacterial activity against 138 isolates with omadacycline MIC ≥ 4mg/liter (MIC_90_, 8 mg/liter). Tigecycline has shown similar antibacterial activity against the 138 tested isolates with MIC_90_ of 4 mg/liter. There were three tested isolates with omadacycline MIC at 16 mg/liter (KBP-7072 at 0.5, 2, and 2 mg/liter, respectively; tigecycline at 2, 8, and 8 mg/liter, respectively) and one isolate with omadacycline MIC at 32 mg/liter (KBP-7072 at 4 mg/liter; tigecycline at 16 mg/liter). In addition, these tested isolates were all resistant to ceftazidime, piperacillin-tazobactam, levofloxacin, and 99.3% resistant to imipenem, 98.6% to meropenem, 93.5% to doxycycline, 92.8% to gentamicin, 96.4% to levofloxacin, and 24.6% to minocycline (Table 5).

**TABLE 5:** *In vitro* activities of KBP-7072 and comparators against 138 omadacycline MIC ≥ 4 mg/liter A. baumannii isolates

Antimicrobial agents	MIC (mg/liter)				R%	S%
	MIC ranges	MIC_50_	MIC_90_	Mode		
KBP-7072	0.5–4	0.5	1	0.5		
Omadacycline	4–32	4	8	4		
Tigecycline	0.5–16	2	4	2		
Doxycycline	1–>128	64	64	64	93.5	5.8
Minocycline	0.5–64	4	16	4	24.6	50.7
Gentamicin	1–>128	>128	>128	>128	92.8	6.5
Ceftazidime	64–>128	128	>128	>128	100	0
Imipenem	2–>128	128	>128	128	99.3	0.7
Meropenem	2–>128	64	128	64	98.6	0.7
Piperacillin-tazobactam	128–>128	>128	>128	>128	100	0
Levofloxacin	4–128	16	64	8	96.4	0
Colistin	0.125–2	0.5	1	0.5	0	100

## DISCUSSION

A. baumannii isolate is one kind of the leading cause of nosocomial infections throughout the world. The surveillance results of 54 tertiary hospitals of China Antimicrobial Surveillance Network (CHINET) in 2021 showed that the isolation rate of A. baumannii among all clinical strains ranked fifth (accounting for 7.62%) (https://www.chinets.com/Data/AntibioticDrugFast). The resistance rate of A. baumannii to meropenem and imipenem has exceeded 65% since 2015. As observed in this study, 69.4% of A. baumannii isolates (372/536) were resistant to carbapenem antibiotics, which was consistent with the increasing tendency of CHINET (https://www.chinets.com/Data/GermYear). Similar to the results of CHINET surveillance, approximately 45% of all global A. baumannii isolates are considered as multidrug-resistant, in which the resistance rate is over 90% in Turkey and Greece, and 60% in the United States, Latin America, and the Middle East (5), respectively. Owing to the characteristics of multidrug-resistance or extensively drug-resistance, the infections caused by A. baumannii isolates were usually associated with high mortality, particularly in the bloodstream and central nervous system infections (9). An increasing trend was observed in the mortality of patients infected with A. baumannii from a 10-year prospective multicenter study in hospitalized patients with bloodstream infection (13).

As the priority pathogens list for research and development of new antibiotics by WHO suggests, new therapeutic development is urgently needed because few antibiotics are available for treating infections caused by CRAB isolates. To date, some new drugs were developed to combat these intractable pathogens, including cefiderocol, sulbactam-durlobactam, and cefepime-zidebactam (14). Several studies have demonstrated cefiderocol good *in vitro* activity against multidrug-resistant A. baumannii isolates (15, 16). Cefiderocol time-dependent *in vivo* efficacy and various preclinical infection models have proved that cefiderocol is efficacious against CRAB isolates, which is predicted by its *in vitro* activity and supported by a reliable PK/PD profile (17–19). Sulbactam-durlobactam had excellent *in vitro* potency against A. baumannii isolates (20, 21). Cefepime-zidebactam also has shown good *in vitro* and *in vivo* antibacterial activity against A. baumannii isolates (22, 23). Whereas these new antimicrobial agents have not been approved in the market of China.

Currently, polymyxins (colistin and polymyxin B) and tigecycline are the last-resort antibiotics for the treatment of infection caused by CRAB isolates. Although colistin has shown well *in vitro* antibacterial activity against CRAB isolates with 99.4% susceptibility in this study and other reports (84.6% to 92.8% susceptibility), (10, 24–26), clinical and PK/PD data demonstrate colistin and polymyxin B have limited clinical efficacy and combination with one or more active antimicrobial agents should be used. Several studies have demonstrated that colistin monotherapy against A. baumannii isolates is not inferior to colistin-based or meropenem combination therapy but has greater nephrotoxicity. (27–29). The emergence of tetracycline resistance determinants *tet(X3)*, *tet(X4)*, and *tet(X5)* in A. baumannii isolates is also worrisome because these genes confer tigecycline resistance, which could inactivate all tetracyclines, including tigecycline and newly U.S. Food and Drug Administration approved eravacycline and omadacycline, and will probably increase more intractable severe infections caused by CRAB isolates in the future (30, 31). Moreover, the correlation between *tet* genes and KBP-7072 is unclear and needs further research. The efficacy of tigecycline in treating CRAB isolates infections also remains debatable, due to its unfavorable pharmacokinetics in the blood and the lung (32). A high dose regimen of tigecycline has been proved efficient in the treatment of hospital-acquired pneumonia and ventilator-associated pneumonia, and the toxicity should be closely monitored because the cases with a decrease in plasma fibrinogen concentration and severe coagulopathy have been reported (33–38). As there are few drugs available in treating A. baumannii isolates infections, we urgently need new agents to combat intractable pathogens with reliable PK/PD.

This study demonstrated that KBP-7072 has active *in vitro* antibacterial activity against 536 A. baumannii isolates (MIC_50/90_, 0.5/1 mg/liter) as supplementary of KBP-7072 pharmacodynamics data in China, which were consistent with the results of the study reported in 2020 that KBP-7072 showed excellent *in vitro* activity against 531 geographically diverse A. baumannii isolates (MIC_50/90_, 0.25/1 mg/liter) collected from North America, Europe, Latin America, and Asia-Pacific (10). In this study, KBP-7072 was significantly superior to other comparators like β-lactams, fluoroquinolone, and aminoglycoside. KBP-7072 was equally active to colistin, outperformed other tetracycline-class comparators against carbapenem-resistant isolates, and maintained activity against ESBL- and MBL-producing isolates (10). In conclusion, KBP-7072 is a potential alternative agent for the treatment of infections caused by A. baumannii isolates, including carbapenem-resistant isolates.

## MATERIALS AND METHODS

### Clinical strains.

A total of 536 nonduplicate A. baumannii isolates was collected from 20 hospitals in 13 provinces and cities in China between January 2018 and December 2019. These A. baumannii isolates were isolated from sputum (69.6%), bronchial alveolar lavage fluid (4.3%,), blood (6.9%,), secreta (4.5%), urine (3.2%), pleural fluid (2.8%), cerebrospinal fluid (2.1%), ascites (1.9%), pus (1.3%), bile (0.9%), catheter (0.4%), drainage (0.4%), aseptic body fluid (0.4%), and other sources (1.5%). Species identification was confirmed by matrix-assisted laser desorption/ionization time of flight mass spectrometry (MALDI-TOF/MS) system (bioMérieux, France).

### Antimicrobial susceptibility testing.

The antimicrobial susceptibility testing of KBP-7072 and comparators was performed utilizing the broth microdilution method according to the Clinical and Laboratory Standards Institute (CLSI) M07 (39). Minimum inhibitory concentrations (MICs) of KBP-7072, omadacycline, tigecycline, doxycycline, minocycline, gentamicin, ceftazidime, imipenem, meropenem, piperacillin-tazobactam, levofloxacin, and colistin were determined. All analyses were performed using WHONET software (version 5.6). Quality control and interpretation of the results were performed according to 2020 CLSI breakpoints for all agents except for the colistin CLSI guideline (40). Colistin MICs were interpreted using 2020 EUCAST MIC breakpoints (susceptible, ≤2 mg/liter; resistant, >2 mg/liter) (http://www.eucast.org).
